# Long-term outcomes of periprocedural coronary dissection and perforation for patients undergoing percutaneous coronary intervention in a Japanese multicenter registry

**DOI:** 10.1038/s41598-023-47444-7

**Published:** 2023-11-20

**Authors:** Toshiki Kuno, Takanori Ohata, Ryo Nakamaru, Mitsuaki Sawano, Masaki Kodaira, Yohei Numasawa, Ikuko Ueda, Masahiro Suzuki, Shigetaka Noma, Keiichi Fukuda, Shun Kohsaka

**Affiliations:** 1 Division of Cardiology, Montefiore Medical Center, Albert Einstein College of Medicine, 111 East 210Th St, New York, NY 10467–2401 USA; 2 Division of Cardiology, Jacobi Medical Center, New York, USA; 3 Department of Cardiology, Keio University School of Medicine, Tokyo, Japan; 4 Department of Healthcare Quality Assessment, The University of Tokyo, Tokyo, Japan; 5 Center for Outcomes Research and Evaluation, Yale New Haven Hospital, New Haven, USA; 6 Department of Cardiology, Japanese Red Cross Ashikaga Hospital, Ashikaga, Japan; 7 Department of Cardiology, National Hospital Organization Saitama Hospital, Wako, Japan; 8 Department of Cardiology, Saiseikai Utsunomiya Hospital, Utsunomiya, Japan

**Keywords:** Cardiology, Interventional cardiology

## Abstract

Long-term outcomes of iatrogenic coronary dissection and perforation in patients undergoing percutaneous coronary intervention (PCI) remains under-investigated. We analyzed 8,721 consecutive patients discharged after PCI between 2008 and 2019 from Keio Cardiovascular (KiCS) PCI multicenter prospective registry in the Tokyo metropolitan area. Significant coronary dissection was defined as persistent contrast medium extravasation or spiral or persistent filling defects with complete distal and impaired flow. The primary outcome was a composite of all-cause death, acute coronary syndrome, heart failure, bleeding, stroke requiring admission, and coronary artery bypass grafting two years after discharge. We used a multivariable Cox hazard regression model to assess the effects of these complications. Among the patients, 68 (0.78%) had significant coronary dissections, and 61 (0.70%) had coronary perforations at the index PCI. Patients with significant coronary dissection had higher rates of the primary endpoint and heart failure than those without (25.0% versus 14.3%, *P* = 0.02; 10.3% versus 4.2%, *P* = 0.03); there were no significant differences in the primary outcomes between the patients with and without coronary perforation (i.e., primary outcome: 8.2% versus 14.5%, *P* = 0.23) at the two-year follow-up. After adjustments, patients with coronary dissection had a significantly higher rate of the primary endpoint than those without (HR 1.70, 95% CI 1.02–2.84; *P* = 0.04), but there was no significant difference in the primary endpoint between the patients with and without coronary perforation (HR 0.51, 95% CI 0.21–1.23; *P* = 0.13). For patients undergoing PCI, significant coronary dissection was associated with poor long-term outcomes, including heart failure readmission.

## Introduction

Significant iatrogenic coronary dissection and perforation are important technical complications that can occur during percutaneous coronary intervention (PCI). These complications are often associated with wiring techniques, increased balloon pressurization, use of inappropriately large balloons or stents, especially in patients with complex anatomical features, such as chronic total occlusion or bifurcation, or use of rotational atherectomy during the procedure^1,2^. Although rare, these can have serious consequences, including fatalities^3,4^. Studies have reported poor in-hospital outcomes, including death, owing to these complications^3–7^. However, limited information is available regarding the long-term consequences for patients who survive these complications during hospitalization.

Investigating the outcomes of patients who have experienced procedural complications is crucial because these patients may be particularly concerned about their prognosis. Accurate long-term information is necessary for clinicians so that they can deliver precise and informed guidance to patients in the event of such complications, plan follow-up visits, and implement preventive measures against complications such as heart failure. Although the benefit of studies on long-term effects of coronary dissection and perforation is expected, limited research is available, especially large-scale observational studies that include consecutive all-comer patients. Therefore, this study aimed to assess the relationship of coronary dissection and coronary perforation with long-term outcomes. The study was conducted in accordance with the principles of the 1964 Declaration of Helsinki.

## Methods

### Database

This study was conducted as part of the Japan Cardiovascular Database-Keio Interhospital Cardiovascular Studies (JCD-KiCS) PCI registry, which is a multicenter prospective registry that includes data of consecutive patients who have undergone PCI since 2008 at 15 institutions within the Tokyo metropolitan area. The details of this registry have been published previously^8–13^. The participating hospitals were instructed to document and register patient data from consecutive hospital visits for PCI, using an Internet-based data collection system. The registered data were reviewed for completeness and internal consistency. Therefore, the study was prospectively designed and retrospectively collected: we predetermined the variables to be collected beforehand, and the recording of patient data was conducted on average 2–3 months after the index procedure. Long-term outcomes, including subsequent revascularization, were reviewed at the 2-year mark after PCI.

Quality assurance of the data was achieved through automatic system validation, reporting of data completeness, and education and training of clinical research coordinators who were specifically trained to use the present PCI registry. The senior study coordinator (I.U.) and exclusive onsite auditing by the investigator (S.K.) ensured appropriate registration of each patient. All the participants provided written informed consent. Before the launch of the JCD-KiCS registry, the University Hospital Medical Information Network of Japan (UMIN000004736) provided information regarding its objective for clinical trial registration. The present study was approved by the IRB committee of Keio University (Reference number: 20080073).

### Studied patients

Of the 8,792 consecutive patients registered between September 2008 and December 2017 with 2-year outcomes, we excluded 58 and 13 patients with missing information on sex and long-term outcomes, respectively, resulting in a final cohort of 8,721 patients.

### Definition of outcomes and variables

The clinical variables and outcomes of the JCD-KiCS were aligned using data from the National Cardiovascular Data Registry (CathPCI Registry version 4.1). Significant coronary dissection was defined as persistent contrast medium extravasation or spiral or persistent filling defects with complete distal flow, impaired flow, or total occlusion. Coronary perforation was defined as leakage of contrast medium from the coronary artery into the surrounding tissue or body cavity. In the JCD-KiCS, all major procedural complications (e.g., death, bleeding complications, and cardiac and cerebrovascular events) were recorded by project coordinators, and the details of the procedural complications were adjudicated by an adjudicator according to a pre-defined data dictionary^13^. A second or third adjudicator was consulted in the event of a disagreement between the opinions of a project coordinator and the first adjudicator.

Acute coronary syndrome (ACS) was defined as ST-segment elevation myocardial infarction, non-ST-elevation myocardial infarction, or unstable angina. Stable coronary artery disease was defined as stable angina, previous myocardial infarction, or silent ischemia. Heart failure was defined as a left ventricular ejection fraction ≤ 35% or documentation of heart failure by the attending physician, regardless of the left ventricular ejection fraction^14^. Multivessel disease was defined as two or more major coronary arteries with ≥ 75% stenosis. The estimated glomerular filtration rate was calculated using the Modification of Diet in Renal Disease Equation for Japanese Patients proposed by the Japanese Society of Nephrology^15–17^.

After hospital discharge, we followed the participants to identify hospitalizations for cardiovascular or bleeding events and all-cause deaths via medical records, phone calls, or mail. All follow-up data at 730 days after PCI were collected and recorded using a secure Internet-based electronic data capture system by dedicated clinical research coordinators trained by the primary investigator and project coordinators^18^. The primary outcome was a composite of ACS, heart failure, coronary artery bypass grafting, stroke and bleeding requiring readmission, and all-cause death. The secondary outcome was a component of the primary outcome. Our captured endpoints were aligned with the NCDR CathPCI registry^17^. Regarding the capture of the revascularization procedure, the intensive utilization of PCI in Japan, involving routine follow-up angiograms until around 2015, led us to incorporate CABG alone as a revascularization endpoint. Our previous collaboration with NCDR CathPCI has indicated that the link between unplanned coronary revascularization with PCI and higher mortality is relatively less significant compared to the association with ACS^19^. Thus, our incorporation of CABG alone as a revascularization procedure can be considered an effort to address and alleviate these potential biases.

### Statistical analyses

Continuous variables are presented as mean ± standard deviation or median (interquartile range), as appropriate, for data distribution. Categorical variables are expressed as percentages. Changes in continuous variables from the baseline were evaluated using Student’s *t*-test or the Mann–Whitney U test. Χ^2^ or Fisher’s exact test was used to analyze categorical variables^13^.

To analyze the long-term outcomes, we utilized Kaplan–Meier estimates and constructed a multivariable Cox proportional hazard model for the primary endpoint, considering the impact of coronary dissection and perforation. The following variables were included in the analysis: age, sex, body mass index, diabetes, hypertension, dialysis, prior myocardial infarction, prior heart failure, indications for PCI, PCI lesions, estimated glomerular filtration rate, hemoglobin level, and puncture site.

All statistical calculations and analyses were performed using R software v. 4.2.2 (R Foundation for Statistical Computing, Vienna, Austria), and p < 0.05 was considered statistically significant.

## Results

In our cohort of 8,721 patients, the mean age of the patients was 68.3 ± 11.3 years. Among the studied patients, 68 (0.78%) had significant coronary dissections, and 61 (0.70%) had coronary perforations. Baseline characteristics and in-hospital and long-term outcomes were compared between the patients with and without coronary dissection and between those with and without coronary perforations (Tables 1, 2, 3, 4).

**Table 1:** Baseline characteristics of patients with dissection and those without.

	Patients without dissection (N = 8653)	Patients with dissection (N = 68)	*P* value
Age	69.00 [61.00, 76.00]	68.00 [61.00, 75.00]	0.524
Male	6796 (78.5)	42 (61.8)	0.001
Body mass index (kg/m^2^)	23.95 [21.89, 26.19]	23.44 [22.15, 26.04]	0.833
Hemoglobin (g/dL)	12.40 [11.00, 13.60]	11.70 [10.10, 12.90]	0.002
eGFR (mL/min./1.73m^2^)	62.35 [48.52, 74.47]	58.67 [43.23, 72.14]	0.088
Smoking	3008 (34.8)	24 (35.3)	1
Previous myocardial infarction	1428 (16.5)	13 (19.1)	0.679
Previous heart failure	688 (8.0)	7 (10.3)	0.627
Diabetes mellitus	3334 (38.5)	23 (33.8)	0.503
Cerebrovascular disease	732 (8.5)	8 (11.8)	0.45
Peripheral artery disease	741 (8.6)	7 (10.3)	0.772
Chronic lung disease	263 (3.0)	7 (10.3)	0.002
Hypertension	6501 (75.1)	49 (72.1)	0.658
Dyslipidemia	5547 (64.2)	40 (58.8)	0.43
Dialysis	314 (3.6)	0 (0.0)	0.203
Previous PCI	1805 (20.9)	12 (17.6)	0.617
Previous coronary bypass	434 (5.0)	4 (5.9)	0.962
Heart failure on admission	1030 (11.9)	14 (20.6)	0.044
Cardiogenic shock on admission	287 (3.3)	2 (2.9)	1
Cardiopulmonary arrest			
on admission	161 (1.9)	3 (4.4)	0.274
Puncture site			0.016
Femoral artery approach	4632 (53.6)	48 (70.6)	
Radial artery approach	3886 (45.0)	20 (29.4)	
Brachial artery approach	126 (1.5)	0 (0.0)	
Significant lesions			
Right coronary artery	4304 (49.7)	39 (57.4)	0.259
Left main	720 (8.3)	5 (7.4)	0.946
Left anterior descending artery	6350 (73.4)	47 (69.1)	0.512
Left circumflex artery	3853 (44.5)	20 (29.4)	0.017
Multivessel disease	4995 (57.7)	36 (52.9)	0.501
Culprit lesions			
Right coronary artery	2733 (31.6)	30 (44.1)	0.037
Left main	326 (3.8)	3 (4.4)	1
Left anterior descending artery	4611 (53.3)	37 (54.4)	0.95
Left circumflex artery	1727 (20.0)	8 (11.8)	0.125
Use of intra-aortic balloon pump	476 (5.5)	12 (17.6)	< 0.001
PCI indication			0.579
ST-elevation myocardial infarction	2298 (26.6)	18 (26.5)	
UA/NSTEMI	2230 (25.8)	20 (29.4)	
Elective	4083 (47.2)	29 (42.6)	
PCI urgency			0.956
Salvage	108 (1.2)	1 (1.5)	
Emergent	2198 (25.4)	16 (23.5)	
Urgent	1910 (22.1)	14 (20.6)	
Elective	4433 (51.3)	37 (54.4)	
Chronic total occlusion	453 (5.2)	8 (11.8)	0.034
Bifurcation lesion	2215 (25.6)	26 (38.2)	0.025
Type C lesion	2524 (29.2)	24 (35.3)	0.331
Use of rotational atherectomy	255 (2.9)	2 (2.9)	1
Use of intravascular ultrasound	7252 (83.8)	56 (82.4)	0.873
Drug eluting stent	6225 (71.9)	41 (60.3)	0.046
Bare metal stent	1589 (18.4)	19 (27.9)	0.061
Left ventricular ejection fraction*	60.00 [50.00, 68.00]	58.50 [50.00, 68.00]	0.814

PCI, percutaneous coronary intervention; UA/NSTEMI, unstable angina/non-ST-elevation myocardial infarction.

Data are presented as the mean ± standard deviation, number (%), and number [interquartile range].

*46.8% of patients had missing values of left ventricular ejection fraction; 29.4% of patients with coronary dissection had missing values of left ventricular ejection fraction.

**Table 2:** In-hospital and long-term outcomes of patients with dissection and those without.

	Patients without dissection (N = 8653)	Patients with dissection (N = 68)	*P* value
In-hospital outcomes			
All complications	584 (6.7)	68 (100.0)	< 0.001
Coronary perforation	59 (0.7)	2 (2.9)	0.135
Myocardial infarction	92 (1.1)	6 (8.8)	< 0.001
Cardiogenic shock	96 (1.1)	3 (4.4)	0.047
Heart failure	135 (1.6)	4 (5.9)	0.019
Cerebral infarction	22 (0.3)	0 (0.0)	1
New induction of dialysis	53 (0.6)	0 (0.0)	1
Cardiac tamponade	18 (0.2)	1 (1.5)	0.358
Transfusion	148 (1.7)	6 (8.8)	< 0.001
Bleeding (all types)	195 (2.3)	6 (8.8)	0.001
Puncture site bleeding	53 (0.6)	2 (2.9)	0.099
Puncture site hematoma	50 (0.6)	0 (0.0)	1
Peritoneal bleeding	8 (0.1)	0 (0.0)	1
Gastrointestinal bleeding	19 (0.2)	1 (1.5)	0.381
Genitourinary bleeding	6 (0.1)	0 (0.0)	1
Intracranial hemorrhage	3 (0.0)	0 (0.0)	1
Other bleeding	73 (0.8)	4 (5.9)	< 0.001
Long-term outcomes requiring readmissions			
Primary endpoint	1267 (14.6)	17 (25.0)	0.026
Death	381 (4.4)	5 (7.4)	0.378
Acute coronary syndrome	313 (3.6)	3 (4.4)	0.981
Heart failure	364 (4.2)	7 (10.3)	0.03
Coronary artery bypass	105 (1.2)	3 (4.4)	0.068
Bleeding	222 (2.6)	1 (1.5)	0.854
Stroke	143 (1.7)	1 (1.5)	1

**Table 3:** Baseline characteristics of patients with perforation and those without.

	Patients without perforation N = 8,660	Patients with perforation N = 61	*P* value
Age	69.00 [61.00, 76.00]	72.00 [65.00, 77.00]	0.277
Male	6794 (78.5)	44 (72.1)	0.299
Body mass index (kg/m^2^)	23.94 [21.89, 26.18]	24.36 [22.29, 26.65]	0.453
Hemoglobin (g/dL)	12.40 [11.00, 13.60]	11.70 [10.35, 12.75]	0.003
eGFR (mL/min./1.73m^2^)	62.35 [48.52, 74.47]	60.12 [46.38, 71.85]	0.234
Smoking	3012 (34.8)	20 (32.8)	0.842
Previous myocardial infarction	1424 (16.4)	17 (27.9)	0.026
Previous heart failure	687 (7.9)	8 (13.1)	0.211
Diabetes mellitus	3336 (38.5)	21 (34.4)	0.601
Cerebrovascular disease	732 (8.5)	8 (13.1)	0.284
Peripheral artery disease	732 (8.5)	8 (13.1)	0.284
Chronic lung disease	268 (3.1)	2 (3.3)	1
Hypertension	6505 (75.1)	45 (73.8)	0.925
Dyslipidemia	5551 (64.2)	36 (59.0)	0.483
Dialysis	311 (3.6)	3 (4.9)	0.834
Previous PCI	1801 (20.8)	16 (26.2)	0.377
Previous coronary bypass	430 (5.0)	8 (13.1)	0.009
Heart failure on admission	1031 (11.9)	13 (21.3)	0.04
Cardiogenic shock on admission	288 (3.3)	1 (1.6)	0.708
Cardiopulmonary arrest on admission	163 (1.9)	1 (1.6)	1
Puncture site			< 0.001
Femoral artery approach	4634 (53.6)	46 (75.4)	
Radial artery approach	3894 (45.0)	12 (19.7)	
Brachial artery approach	123 (1.4)	3 (4.9)	
Significant lesions			
Right coronary artery	718 (8.3)	7 (11.5)	0.506
Left main	4310 (49.8)	33 (54.1)	0.585
Left anterior descending artery	6346 (73.3)	51 (83.6)	0.094
Left circumflex artery	3840 (44.3)	33 (54.1)	0.162
Multivessel disease	4987 (57.6)	44 (72.1)	0.031
Culprit lesions			
Right coronary artery	2747 (31.7)	16 (26.2)	0.435
Left main	327 (3.8)	2 (3.3)	1
Left anterior descending artery	4614 (53.3)	34 (55.7)	0.799
Left circumflex artery	1719 (19.8)	16 (26.2)	0.279
Use of intra-aortic balloon pump	481 (5.6)	7 (11.5)	0.084
PCI indication			0.043
ST-elevation myocardial infarction	2303 (26.6)	13 (21.3)	
UA/NSTEMI	2241 (25.9)	9 (14.8)	
Elective	4074 (47.0)	38 (62.3)	
PCI urgency			0.146
Salvage	108 (1.2)	1 (1.6)	
Emergent	2204 (25.5)	10 (16.4)	
Urgent	1914 (22.1)	10 (16.4)	
Elective	4430 (51.2)	40 (65.6)	
Chronic total occlusion	446 (5.2)	15 (24.6)	< 0.001
Bifurcation lesion	2219 (25.6)	22 (36.1)	0.087
Type C lesion	2516 (29.1)	32 (52.5)	< 0.001
Use of rotational atherectomy	250 (2.9)	7 (11.5)	< 0.001
Use of intravascular ultrasound	7260 (83.8)	48 (78.7)	0.362
Drug eluting stent	6226 (71.9)	40 (65.6)	0.342
Bare metal stent	1601 (18.5)	7 (11.5)	0.214
Left ventricular ejection fraction*	60.00 [50.00, 68.00]	60.00 [47.25, 67.50]	0.693

PCI, percutaneous coronary intervention; UA/NSTEMI, unstable angina/non-ST-elevation myocardial infarction.

Data are presented as the mean ± standard deviation, number (%), and number [interquartile range].

*: 46.8% of patients had missing values of left ventricular ejection fraction; 31.1% of patients with coronary dissection had missing values of left ventricular ejection fraction.

**Table 4:** In-hospital and long-term outcomes of patients with perforation and those without.

	Patients without perforation N = 8,660	Patients with perforation N = 61	*P* value
In-hospital outcomes			
All complications	591 (6.8)	61 (100.0)	< 0.001
Coronary dissection	66 (0.8)	2 (3.3)	0.135
Myocardial infarction	97 (1.1)	1 (1.6)	1
Cardiogenic shock	94 (1.1)	5 (8.2)	< 0.001
Heart failure	139 (1.6)	0 (0.0)	0.628
Cerebral infarction	22 (0.3)	0 (0.0)	1
New induction of dialysis	52 (0.6)	1 (1.6)	0.831
Cardiac tamponade	15 (0.2)	4 (6.6)	< 0.001
Transfusion	149 (1.7)	5 (8.2)	0.001
Bleeding (all types)	199 (2.3)	2 (3.3)	0.936
Puncture site bleeding	55 (0.6)	0 (0.0)	1
Puncture site hematoma	50 (0.6)	0 (0.0)	1
Peritoneal bleeding	8 (0.1)	0 (0.0)	1
Gastrointestinal bleeding	20 (0.2)	0 (0.0)	1
Genitourinary bleeding	6 (0.1)	0 (0.0)	1
Intracranial hemorrhage	3 (0.0)	0 (0.0)	1
Other bleeding	75 (0.9)	2 (3.3)	0.187
Long-term outcomes requiring readmissions			
Primary endpoint	1253 (14.5)	5 (8.2)	0.228
Death	370 (4.3)	1 (1.6)	0.486
Acute coronary syndrome	308 (3.6)	1 (1.6)	0.646
Heart failure	365 (4.2)	3 (4.9)	1
Coronary artery bypass	107 (1.2)	1 (1.6)	1
Bleeding	218 (2.5)	1 (1.6)	0.979
Stroke	141 (1.6)	0 (0.0)	0.62

A significantly lower proportion of males had coronary dissections, and the proportions of chronic lung disease, heart failure at admission, femoral artery approach, right coronary artery culprit vessel, use of an intra-aortic balloon pump, and chronic total occlusion, and bifurcation were higher among those with coronary dissections than among those without (Table 1). There were no significant differences in left ventricular ejection fraction between patients with coronary dissection and those without. Additionally, patients with coronary dissection had higher rates of post-PCI myocardial infarction, cardiogenic shock, heart failure, and transfusion than those without (Table 2).

The proportions of prior myocardial infarction, coronary bypass, heart failure on admission, femoral artery approach, multivessel disease, chronic total occlusion, type C lesions, and use of rotational atherectomy were higher among the patients with coronary perforations than among those without (Table 3). Moreover, the patients with coronary perforations had higher rates of post-PCI cardiogenic shock, cardiac tamponade, and transfusion, however, only 6.6% of patients with coronary perforation suffered from cardiac tamponade (Table 4).

Importantly, the patients with significant coronary dissections had higher rates of the primary endpoint (25.0% versus 14.3%, *P* = 0.02) and heart failure requiring readmission (10.3% versus 4.2%, *P* = 0.03) than those without, and there was a marginal difference in the rate of coronary bypass (4.4% vs. 1.2%, *P* = 0.068) between the former and latter. Interestingly, there were no significant differences in the primary and secondary outcomes between the patients with and without coronary perforations (primary outcome: 8.2% vs. 14.5%, *P* = 0.23).

**Figure 1:**
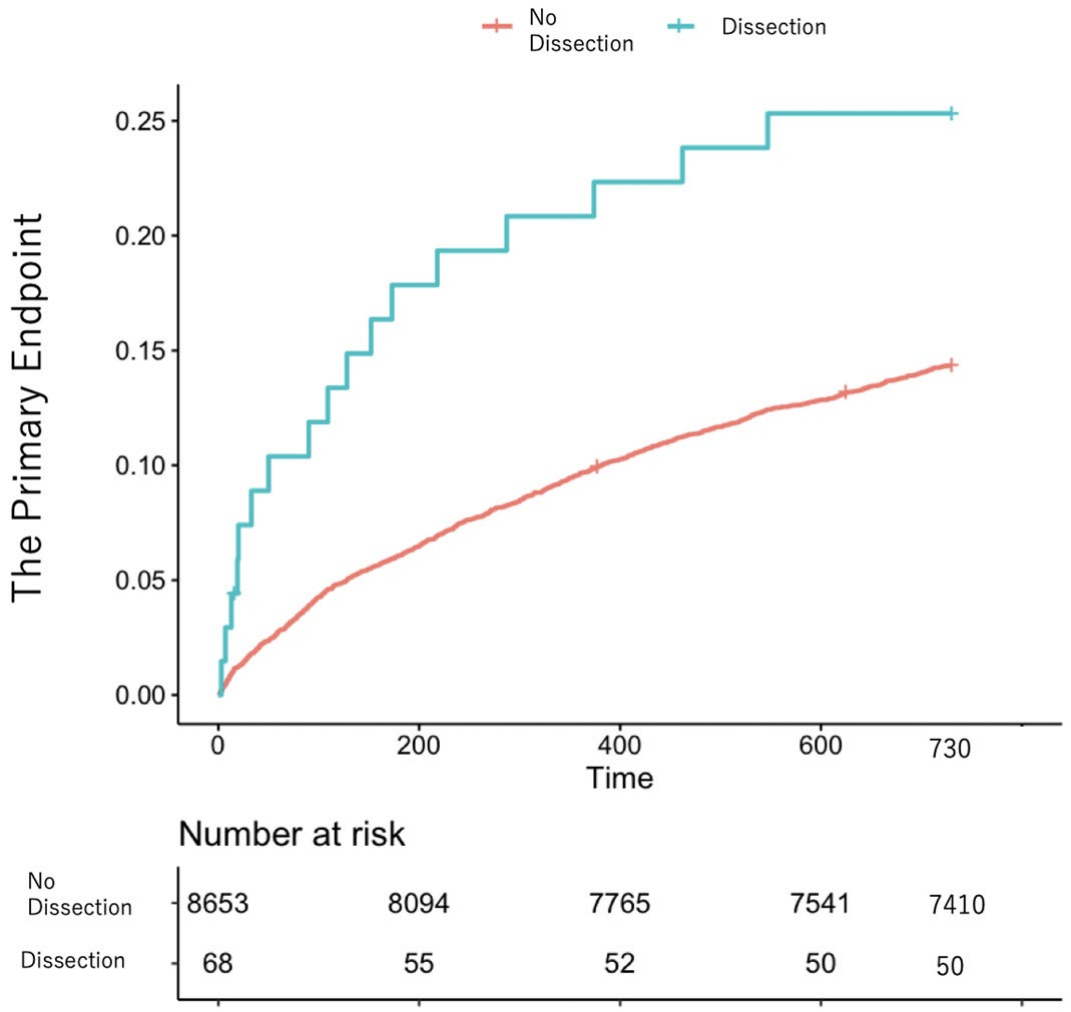
Kaplan–Meier curve of long-term outcomes (primary endpoint) for patients with and without coronary dissection.

**Figure 2:**
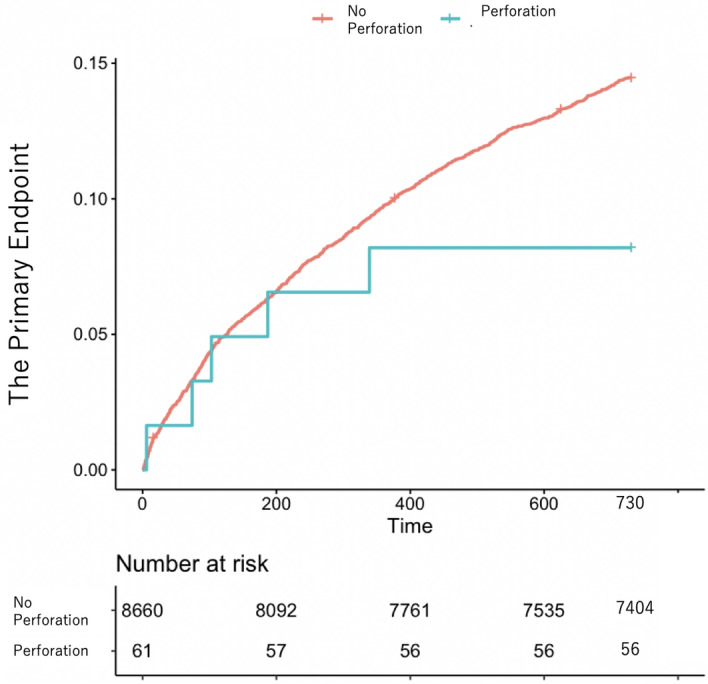
Kaplan–Meier curve of long-term outcomes (primary endpoint) for patients with and without coronary perforation.

The Kaplan–Meier curves of the primary endpoint for patients with and without coronary dissections and coronary perforations are shown in Figs. 1 and 2. The patients with coronary dissection had significantly higher rates of the primary endpoint than those without (unadjusted hazard ratio [HR]: 1.98, 95% confidence interval [CI]: 1.22–3.19; *P* = 0.005) at the 2-year follow-up, but there was no significant difference in the primary endpoint between those with and without coronary perforations (unadjusted HR 0.56, 95% CI 0.23–1.34; *P* = 0.19).

After adjusting the multivariable Cox proportional hazard risk model for other variables, patients with coronary dissections showed a significantly higher rate of the primary endpoint than those without (adjusted HR 1.70, 95% CI 1.02–2.84; *P* = 0.04) at the 2-year follow-up, but there was no significant difference in the primary endpoint rate between those with and without coronary perforation (adjusted HR 0.51, 95% CI 0.21–1.23; *P* = 0.13).

## Discussion

The salient findings of our study are as follows: In a cohort of 8,721 PCI patients with a 2-year follow-up, significant coronary dissection (0.78% of patients) was associated with notable differences in the baseline characteristics, procedural details, and outcomes, including increased rates of post-PCI myocardial infarction, cardiogenic shock, and transfusion. Patients with significant coronary dissection also had higher rates of primary endpoints and heart failure requiring readmission than those without. However, there were no significant differences in the outcomes between the patients with and without coronary perforation. The Kaplan–Meier curves indicated a higher rate of the primary endpoint for patients with coronary dissection than for those without, confirmed by the multivariable Cox proportional hazard risk model. No difference was observed between the patients with and without coronary perforation.

Very limited long-term data are available on the impact of significant coronary dissection in all-comer PCI registries, with only one report exploring iatrogenic catheter-induced coronary dissection. However, this study focused only on catheter-induced coronary dissection of the ostium of the coronary artery. It is crucial to avoid significant coronary dissection during PCI and recognize known risk factors such as female sex, complex PCI such as chronic total occlusion, long lesions and calcification, and the use of a relatively large balloon size^4,20^. Female patients tend to have smaller coronary arteries, and this may cause procedure-related coronary dissections frequently.

Our findings revealed that patients with coronary dissection experienced worse outcomes, including high rates of primary endpoint and heart failure readmissions and a tendency to require coronary bypass and medication adjustment with close monitoring post-discharge. Interestingly, patients with coronary dissection did not have lower left ventricular ejection fraction compared to those without. We hypothesized that residual coronary dissection may cause myocardium ischemia, which results in diastolic dysfunction^21^. Since we demonstrated that patients with significant coronary dissection had a higher risk of heart failure readmission, they may need to have an appointment within 1 week after discharge or diuresis adjustment with a heart failure specialist to decrease the risk of readmission for heart failure^22^.

Coronary perforation is a serious complication that can lead to pericardial effusion, causing cardiac tamponade, which can be fatal if not treated promptly^23–25^. The long-term consequences of coronary perforation are not well understood, but studies on patients with chronic total occlusions have shown that perforation has a legacy effect on mortality, with an odds ratio for 12-month mortality of 1.60 for perforation survivors compared with that for those without perforation^24^. However, these data are limited to patients with chronic total occlusions, who could have higher risk profiles than other PCI patients because coronary perforation, especially collateral circulation, may worsen the myocardium supply (i.e., due to coil embolization). We found an overall decline in mortality related to coronary artery perforation over time^26^ and the risk of additional adverse events in the long term may be low, as suggested in our study. Moreover, our all-comer PCI registry showed that only a quarter of patients with coronary perforation had chronic total occlusion, and only 6.6% of the patients had developed cardiac tamponade related to coronary perforation, and this could explain why they did not experience poor long-term outcomes.

Compared to coronary dissection and perforation, which are less frequently encountered procedural complications, other complications, such as bleeding or acute kidney injury, have been extensively studied and shown to impact both in-hospital and long-term mortality^27,28^. For example, PCI-related bleeding complications are relatively common and well studied^29–31^. These complications can affect not only in-hospital mortality but also long-term mortality^27,32^. Given these poor outcomes, we implemented bleeding avoidance strategies, such as the transradial approach, to decrease the risk of bleeding complications.

Our study had several limitations. First, this was an observational study, and our analysis could not be adjusted for unmeasured confounders. Second, in our registry, the follow-up survey focused only on clinically driven events. Therefore, subsequent revascularization was retrospectively reviewed, and some revascularization events may not have been captured, especially, in cases of transfer to institutions outside of the JCD‐KiCS network. Third, we did not adjust for left ventricular ejection fraction because almost half of the whole patients and a third of patients with coronary dissection or perforation did not have information on left ventricular ejection fraction; however, left ventricular ejection fractions were similar between patients with and without coronary perforation or dissection. Fourth, we did not collect data on the treatment for coronary dissection or perforation (i.e., covered stent, coil embolization, or cutting balloon) that may have affected future events, such as revascularization^6,33^. In addition, we did not collect data on the detailed significance of coronary perforation (i.e., Ellis classification)^34^. Previous studies have suggested a correlation between the Ellis classification and long-term outcomes or the requirement for covered stents^33^. While our definition of coronary perforation aligns with other internationally recognized registries, the evaluation of the degree of perforation may be necessary in future studies to elucidate its precise impact on long-term implications. However, only a few patients with coronary perforation suffered from cardiac tamponade (6.6%), which may reflect a better prognosis in patients with coronary perforation than expected.

In conclusion, in patients undergoing PCI, significant coronary dissection was associated with poor long-term outcomes, but not in those with coronary perforation. Our long-term data on coronary dissection and perforation can provide valuable insights for physicians who follow patients in the post-discharge setting and promote the use of appropriate procedures (such as the use of intravascular imaging) to eliminate the risk of fatal complications.

## Data Availability

The datasets used and/or analyzed during the current study available from the corresponding author on reasonable request.

## Abbreviations

ACS: Acute coronary syndrome
JCD-KiCS: Japan cardiovascular database-Keio Interhospital cardiovascular studies
PCI: Percutaneous coronary intervention
